# “Getting out of downtown”: a longitudinal study of how street-entrenched youth attempt to exit an inner city drug scene

**DOI:** 10.1186/s12889-017-4313-9

**Published:** 2017-05-02

**Authors:** Rod Knight, Danya Fast, Kora DeBeck, Jean Shoveller, Will Small

**Affiliations:** 1 Faculty of Health Sciences, Simon Fraser University, 8888 University Drive, Burnaby, BC V5A 1S6 Canada; 2 British Columbia Centre for Excellence in HIV/AIDS, 608-1081 Burrard Street, Vancouver, BC V6Z 1Y6 Canada; 3 School of Population and Public Health, University of British Columbia, 2206 East Mall, Vancouver, V6T 1Z3 Canada; 4 Department of Medicine, University of British Columbia, 2775 Laurel Street, 10th Floor, Vancouver, BC V5Z 1M9 Canada; 5 School of Public Policy, Simon Fraser University at Harbour Centre, 515 West Hastings Street, Vancouver, BC V6B 5K3 Canada; 6 British Columbia Centre on Substance Use, British Columbia Centre for Excellence in HIV/AIDS, St. Paul’s Hospital, 608-1081 Burrard Street, Vancouver, BC V6Z 1Y6 Canada

**Keywords:** Canada, Youth, Addiction, Drug scene, Street entrenched

## Abstract

**Background:**

Urban drug “scenes” have been identified as important risk environments that shape the health of street-entrenched youth. New knowledge is needed to inform policy and programing interventions to help reduce youths’ drug scene involvement and related health risks. The aim of this study was to identify how young people envisioned exiting a local, inner-city drug scene in Vancouver, Canada, as well as the individual, social and structural factors that shaped their experiences.

**Methods:**

Between 2008 and 2016, we draw on 150 semi-structured interviews with 75 street-entrenched youth. We also draw on data generated through ethnographic fieldwork conducted with a subgroup of 25 of these youth between.

**Results:**

Youth described that, in order to successfully exit Vancouver’s inner city drug scene, they would need to: (a) secure legitimate employment and/or obtain education or occupational training; (b) distance themselves – both physically and socially – from the urban drug scene; and (c) reduce their drug consumption. As youth attempted to leave the scene, most experienced substantial social and structural barriers (e.g., cycling in and out of jail, the need to access services that are centralized within a place that they are trying to avoid), in addition to managing complex individual health issues (e.g., substance dependence). Factors that increased youth’s capacity to successfully exit the drug scene included access to various forms of social and cultural capital operating *outside* of the scene, including supportive networks of friends and/or family, as well as engagement with addiction treatment services (e.g., low-threshold access to methadone) to support cessation or reduction of harmful forms of drug consumption.

**Conclusions:**

Policies and programming interventions that can facilitate young people’s efforts to reduce engagement with Vancouver’s inner-city drug scene are critically needed, including meaningful educational and/or occupational training opportunities, ‘low threshold’ addiction treatment services, as well as access to supportive housing outside of the scene.

## Background

### Introduction

Urban drug “scenes” – broadly defined as inner city areas featuring large drug-using populations and high levels of street-based drug dealing (and often homelessness or unstable housing) – have been identified as important risk environments that shape the health of vulnerable populations, including youth [1, 2]. Previous research indicates that young people (e.g., 30 years of age and under) who use drugs in the context of drug scene entrenchment experience elevated rates of infectious (e.g., HIV, Hepatitis C) and chronic (e.g., diabetes, liver disease) diseases [3–6] amidst intersecting forms of social vulnerability [7–12]. Moreover, young people entrenched in urban drug scenes frequently experience extreme socioeconomic deprivation, homelessness, addiction, mental health issues and involvement in dangerous and criminalized income-generation activities [1, 13–16]. Reducing youths’ drug scene involvement and related health risks is a critical priority for public health intervention.

The investigation of young people’s transitions *into* homelessness, as well as evolving forms of harmful drug use, has been an important focus for research and policy and programming interventions [17, 18]. For example, previous research has identified risk factors associated with youth’s transitions into homelessness, including family dysfunction, experiences of abuse and trauma, various forms of exploitation, poverty, mental health crises, and a lack of culturally appropriate social welfare services [17]. Research from Vancouver, Canada, has identified how transitions into more harmful forms of illicit drug use among youth can be powerfully shaped by geographical transitions between neighbourhoods, and the social, structural and spatial processes that are embedded in particular places [1, 15]. As young people become entrenched in these scenes/milieus across time, transitions into homelessness and more harmful forms of drug use can come to seem increasingly inevitable given the constraints of their everyday lived experience. Indeed, in settings like Vancouver’s Downtown Eastside and Downtown South neighbourhoods, decision-making and transitions related to drug use tend not to be focused so much on whether to take drugs or quit taking drugs, but rather on shifting definitions of “desirable” versus “undesirable” forms of drug use [13].

While there is an established body of research investigating young people’s transitions *into* various forms of drug involvement (e.g., more or less harmful forms of illicit use), as well as various forms of precarious housing and homelessness, far less research has explored how street-entrenched young people think about and enact transitions *away from* and *out of* urban drug scenes. Given the numerous harms and intersecting forms of disadvantage experienced by young people who are drug scene entrenched, new knowledge to inform policy and programming is critically needed to better understand how young people can best be supported to reduce or eliminate their involvement with urban drug scenes. Drawing on a longitudinal study design and in-depth interviews with street-entrenched young people in Vancouver, Canada, the aim of this study was to identify how young people envision exiting a local, inner-city drug scene in Vancouver, as well as the individual, social and structural factors that shape young people’s trajectories as they attempt to exit this setting.

## Methods

### Study setting

This study is part of a larger program of ethnographic and qualitative research examining young people’s substance use and broader risk trajectories in the context of Vancouver’s inner city, street-based drug scene [1, 13, 19]. This drug scene primarily consists of two distinctive neighbourhoods: the Downtown Eastside and the Downtown South. Our long-term ethnographic research shows that street entrenched young people in our setting frequently move between these two neighbourhoods, as they navigate elaborate social spatial networks and the day-to-day realities of securing basic necessities. Although the Downtown Eastside and the Downtown South are geographically adjacent (and within easy walking distance of each other), they are consistently differentiated according to their history and a number of aspects of place. The Downtown Eastside is widely conceived as Vancouver’s poorest and most ‘run-down’ neighbourhood, and is the site of a large concentration of low-income, government-subsidized housing and numerous services that provide shelter, healthcare, food and harm reduction services to vulnerable populations (including large numbers of people who inject drugs). The Downtown South is a residential and entertainment district characterized by both high- and some low-income housing, as well as numerous entertainment venues (e.g., nightclubs) and other commercial enterprises (e.g., restaurants, shops). Both neighbourhoods are characterized by shadow economies largely propelled by drug dealing, the exchange of stolen/s hand goods, and sex-work activities.

The Downtown South and Downtown Eastside neighbourhoods are also differentiated by substance use patterns, although much overlap exists and it should also be noted that both neighbourhoods are inhabited by a large number of people who *do not* use illicit drugs. While the Downtown Eastside is generally identified with a trade in crack cocaine, cocaine, heroin and prescription opioids bought and sold primarily by older individuals, the Downtown South is generally identified with high rates of crystal methamphetamine sales and use primarily among youth [20]. Although various youth services are now situated in the Downtown South (e.g., a large drop-in centre, a shelter, a community health clinic, a job search service, several outreach programs and mobile health vans), decision makers and advocacy groups continue to struggle to address the “street youth problem” in this setting [14].

### Data collection

We draw on over 150 audio-recorded and transcribed semi-structured interviews with 75 street-entrenched youth conducted in four waves from 2008 to 2016 (see Table 1). These interview waves were embedded within an eight-year program of ethnographic research conducted by the second author (DF), which allows us to further contextualize the findings that emerged from these interview series. This larger program of ethnographic research involved spending hundreds of hours in the places where young people were living, working, sleeping, socializing, and accessing services and systems, documented through written field notes, audio-recordings, and photographs. All interview participants were recruited by DF from the At-Risk Youth Study (ARYS), a prospective cohort of drug-using and street-involved youth in Vancouver. ARYS cohort members are between the ages of 14 and 26 years and self-report the use of illicit drugs other than or in addition to marijuana in the past thirty days, at the time of enrolment. See Wood et al. 2006 for more details on the ARYS cohort [20]. Rather than randomly selecting participants to ensure their “representativeness” of the broader street-entrenched population of youth in Vancouver, we purposefully sampled young people who, by virtue of their lived experiences, had the capacity to reflect on the processes involved in exiting Vancouver’s urban drug scene. This included selecting and recruiting youth with various social identities (e.g., sexual identities; ethno-racial identities; gender identities) and lived experiences (e.g., experiences with: injection drug use; sex work; drug dealing).

**Table 1:** Data collection processes from 2008 to 2016

	Wave 1	Wave 2	Wave 3	Wave 4
Dates	January 2008 to June 2008	July 2009 to April 2010	January 2011 to December 2012	January 2013–May 2016
Data collection techniques	In-depth interviews	In-depth interviews	Intensive ethnographic fieldwork documented through fieldnotes, audio recordings	Intensive ethnographic fieldwork documented through fieldnotes, audio recordings
Research participants	39 (18 men; 21 women)	28 (20 men; 8 women)	25 (16 men; 9 women)	15 (9 men; 5 women; 1 transgender individual)
Age range	16–26 (median age = 22)	14–25 (median age = 21)	20–30 (median = 26)	24–34 (median age = 28)
Ethnicity	65% Caucasian, 25% of Indigenous ancestry, 10% African Canadian	72% Caucasian, 25% of Indigenous ancestry, 3% African Canadian	67% Caucasian, 25% of Indigenous ancestry, 8% African Canadian	60% Caucasian, 33% of Indigenous ancestry, 7% African Canadian
Follow-up from previous waves	N/A	12 from Wave 1	DF had contact with research participants on at least a monthly basis; in addition, 40 more formal, audio recorded semi-structured interviews were conducted during this time	DF had contact with research participants on at least a monthly basis; in addition, 56 more formal, audio recorded semi-structured interviews were conducted during this time
Recruitment process	Facilitated by a youth peer research associate	Facilitated by the staff at the ARYS research office, and Fast’s ongoing relationships with local youth	Facilitated by DF’s ongoing relationships with local youth	Facilitated by DF’s ongoing relationships with local youth
Primary Location	The ARYS research office in the Downtown South	The ARYS research office in the Downtown South	The places where youth were living, socializing and working	The places where youth were living, socializing and working

During interviews, youth reflected on the current nature of their drug scene involvement, and how their involvement had shifted across time as they attempted to navigate drug use, multiple income generation strategies, unstable or undesirable housing, and social relationships with a wide range of actors including friends, romantic partners, “business partners” (in the drug trade), service providers and the police. All of the young people in our sample frequently spoke about their future aspirations, including their plans to reduce their involvement in the scene. Participants reflected on their attempts to enact particular exits across multiple waves of data collection. All participants provided written informed consent to participate in in-depth interviews, and received a twenty to thirty dollar honorarium for each interview (the amount of the honorarium was increased in 2013). The study was undertaken with ethical approval granted by the Providence Healthcare/University of British Columbia Research Ethics Board.

### Data analysis

Audio-recorded interviews were transcribed verbatim, anonymized and checked for accuracy. ATLAS.TI software was used to manage the transcribed interview data. Data analysis was guided by three overarching analytical questions: (1) *How do young people envision exiting the inner city drug scene?*; (2) ‘*What are the critical moments that influence youths’ experiences of “getting out” (*e.g.*, the individual, social, structural, and environmental factors that shape these experiences over time)?’*; and (3) ‘*How are young people’s transitions out of the inner-city drug scene shaped by key periods (*e.g.*, when do they express a desire to “get out” or decrease their involvement with the scene and/or reflect on a plan to do so)*?’

Co-authors DF and RK coded the interviews, whereby data were highlighted and corresponding themes, labels, and notes about interpretations were made within the interview transcripts. We used constant comparative analytical techniques [21], in which we evaluated our emerging analyses in relation to previous research and all of our existing data in the current analysis. Specifically, the individual transcripts were compared and contrasted to identify patterns, as well as shared and divergent understandings identified. Discrepancies between codes and emergent themes were resolved through discussion and re-visiting the raw data during team meetings. Memos were kept by the lead author (RK) to document the analysis process as it developed, and these were discussed with the co-authors throughout the analysis of the data and writing of this article. We also used “verification” strategies to establish rigor in our approach to validating our decisions about the analysis, including the emphasis of responsiveness and openness of all authors to the data in adjusting or revising the codes and presentation of each thematic to result in a consensus-driven representation of our findings. As this process unfolded, we developed a set of codes to identify the broad themes that emerged across the data set (e.g., “exiting and physical and mental health”; “exiting and interpersonal relationships”; “the role of existing policies and programs”). As such, our analysis draws on both a deductive approach, in which findings were compared and contrasted to existing theoretical and empirical literature, as well as an inductive approach, through which we developed our coding schematic. Within our presentation of our analysis, we have sought to be highly contextual and descriptive with regards to describing the extent to which changes have occurred over time with each of our participants. To do so, we have offered an approach to presenting our longitudinal data and analysis in a way that allows us to present across- and within-person variations over time by providing both a data point (i.e., quote from an in-depth interview) along with a descriptive narrative to illustrate to the reader how these changes have transpired over time.

## Results

### Sample characteristics

Our sample of 75 participants frequented many of the same service locations and had likely used drugs together in the parks or alleyways of downtown Vancouver at one time or another. Yet, only a small number were networked in a more enduring sense, usually in the form of a romantic or crime-related partnership. Nevertheless, participants did share a number of common characteristics. The majority of youth grew up in low-income, materially disadvantaged households; approximately three-fifths (*n* = 43) had experienced violence, abuse or neglect in their pasts and slightly less than half (*n* = 33) had a history of government (foster) care. Slightly less than half of participants (*n* = 36) had a history of involvement in the juvenile justice system, and/or had served time in a provincial prison as an adult. Only 15 of 75 participants had graduated from high school or later completed their General Education Diploma. Approximately three-fifths of youth (*n* = 44) indicated that they had been diagnosed with a mental illness by a health professional, and a number of additional participants indicated that they suffered from a self-diagnosed mental health issue.

All but two participants reported being homeless during the course of the study (meaning that they were sleeping outside, “couch surfing” at friends’ or acquaintances’ places, and/or staying at shelters – most often a combination thereof). At some point during the study period, all youth were engaged in drug use practices that they defined as problematic, using terms like “addiction,” “out-of-control” and “self-destructive.” Based on our fieldwork and interview data, these terms consistently referred to drug use which was resulting in physical, psychological and/or emotional problems, whether directly as a result of the chemical properties of a particular substance, or indirectly as a result of the violence and instability that often accompanies drug use in the context of social, spatial and economic marginalization. For the young people who participated in this study, this included the daily use of heroin, crack and/or crystal methamphetamine (meth). Fifty-five youth had used meth at least once in their lives; thirty-three had injected meth. Youth also engaged in varying forms of poly-substance use over time; for example, at any one time, up to half of those youth who reported using meth also reported using crack and/or heroin.

Approximately three-quarters of participants (*n* = 54) received monthly social-assistance (welfare) payments, although this support was rarely enough to cover the cost of living in Vancouver. Most youth were therefore involved in a range of illicit income-generation activities, including street-level drug dealing (*n* = 51), sex work (*n* = 14), and the exchange of stolen and purchased goods (*n* = 27). In sum, the majority of these youth were or had at one time been significantly “entrenched” in Vancouver’s street-based drug scene; they described themselves as being, or recently having been, largely consumed by a daily project of survival “on the streets” – a phrase that in our setting refers to numerous indoor and outdoor settings associated with the drug scene.

### Findings

During the interviews and fieldwork with young people, we asked participants to describe the futures they envisioned for themselves. While a minority of the participants accepted a future in which they would remain entrenched in Vancouver’s inner-city drug scene as ‘inevitable’, being addicted to drugs and dependent on the social service infrastructure, the vast majority of young people expressed a strong desire to exit the scene. As participants described their future aspirations, many indicated that they wanted to exit the scene in order to reconnect with some aspect of their pasts, and they frequently expressed a desire to do so independently and/or with limited supports. For others, an exit from the scene would represent a first step towards obtaining what they often referred to as a “normal life” in Vancouver. In order to successfully exit Vancouver’s inner city drug scene, all of the youth articulated one or more beliefs that they would need to: (a) secure legitimate employment and/or obtain education or occupational training; (b) distance themselves – both physically and socially – from the urban drug scene; and (c) reduce their drug consumption, including through the use of addiction treatment services. In the following sections, we describe the challenges that young people faced in exiting the drug scene and actualizing these visions of a “normal life” in Vancouver. We present quotes from our interview data to illustrate key aspects of our analysis, and provide context about the participants and their attempts to exit by drawing on data derived from both our interview and ethnographic data. All names appearing below are pseudonyms.

#### Challenges finding legal employment and reducing reliance on illegal income generation

Participants described a deep desire to secure “legitimate,” legal employment that was partially or fully removed from the street-based drug scene. There were often dramatic differences between the kinds of jobs that youth envisioned securing and the kinds of employment that they were actually able to obtain in the context of low levels of education (in almost all cases) and a lack of the social and symbolic capital required to obtain employment. While many youth had at least some prior experience working in a variety of labour and service sector jobs, they often found themselves ill-equipped for the job-hunting process, including being unable to answer conventional interview questions from prospective employers. For example, Dave, a 23-year-old young Indigenous man described how, even after securing an interview, he had not been offered the job because of “totally blowing” the interview process:

*They asked me these hard questions that I couldn’t answer, and I totally blew it. Yeah, the simplest questions like* “What do you bring to the table?” *What’s your strength?” And it’s like,* “I thought you were just going to ask me if I knew how to do the job.”

As participants reflected on the practical actions that would be required to take in order to secure some form of legal employment, a number described a need to both address the most unstable aspects of their everyday lives (e.g., the imperative to generate income in the context of addiction and unstable housing), *and* successfully navigate a number of other bureaucratic tasks (e.g., securing welfare payments). As a result of the high level of chaos that characterized their everyday lives on the streets, youth lamented “never seeming to be able to find the time” to prepare resumes, do job searches and fill in job applications. Printed documents of this kind were also difficult for many young people to carry around and store – whether in the context of street-based homelessness or residence in a run down single room occupancy hotel (SRO). Living conditions on the streets and inside SROs also made it difficult for youth to store and maintain clothing that would be appropriate for job interviews.

In spite of the fact that an employment service infrastructure exists in the Downtown South in order to help youth with job searches and preparing job applications and resumes, it is worth noting that a large number of young people insisted that these were tasks they planned and wanted to accomplish on their own. Many of the youth expressed a strong distrust towards “the system” and various aspects of the social service and health infrastructure due to previous experiences (e.g., negative experiences with foster care). However, as they attempted to navigate the multiple steps required in order to enter the legal job market, many young people became confused, in part because the majority of participants had extremely limited computer literacy skills (e.g., did not have the skills to fill out online applications). For example, Lee, a 27-year-old young Indigenous man described how he needed government identification before he could enrol in an alternative job training program that he imagined would improve his chances of getting a “real” job. When asked about how he was going to go about getting his ID, his response revealed some of his confusion in sorting through the logistics of multiple application processes with little support or guidance:

*I wanna go one step at a time. […] The ID would be primary – first. And then look for a job. No, no! Look for a job first, right?* *‘Cause then I could try to get my ID at the same time. But I don*’*t know how that’s going to work, with working and getting it at the same time, it’s gonna be… ‘Cause I’ll be working during the week, and, the hours will be gone. And I don’t know who would want to do that for me, or if I have to do it for myself.*

A number of young people described their attempts to enrol in various training and education programs. Many were not able to do so successfully due to what they perceived to be complex admission requirements (e.g., the need to provide transcripts of previous grades, the need to meet certain course prerequisites), as well as limited opportunities for financial support. Others experienced barriers to completion of these programs upon enrolment. For example, even in the context of alternative school and training programs that are designed for “at-risk” youth, young people could quickly become overwhelmed by the content of lessons, leading some to “give up” and leave the program without approaching the instructor or others for help. Again, many young people lacked the social capital needed to successfully communicate the challenges they were experiencing with teachers and administrators (many of whom would likely have been very receptive to requests for extra help). Moreover, some of the forms of social and symbolic capital that young people had accumulated on the streets and across their lives (e.g., aggressive ways of talking and interacting with others) could be at odds with the kinds of social and symbolic capital that would help them to succeed in job interviews, training, and school programs. Young people were highly cognizant of the mismatch between the kinds of traits (e.g., toughness, aggression, independence) they had cultivated in order to survive and thrive in places like government care homes, the streets of Vancouver, and prison, versus those desired by potential employers and educators. Jordan, a 27-year-old Caucasian man described how his previous life experiences made him more comfortable with the prospect of going to prison than university – despite his desire for a university education:

*My whole family’s been in and out of jail. […] I never went into a real school surrounding. But I did the correspondence [to get his General Educational Development while in jail]. I wish I could’ve went into a real school surrounding. Probably still could. I’m thinking about going to UBC [the University of British Columbia]. But I’d be kind of intimidated of going to UBC. Cause there’s nice people like yourself going there…I’d be more intimidated going to UBC [than jail]. I get really intimidated when I get around normal crowds.*

While youth emphasized their desire to obtain some form of job or education training, it is striking to note that, at the time of writing, none of the participants followed throughout our study completed training and education programs except for those who did so while in a correctional facility.

While relatively few youth managed to do so, securing legal employment outside of the inner-city drug scene represented a critical moment in youth’s transitions out of the scene, as these moments profoundly influenced young people’s trajectories with “getting out” of the urban drug scene. However, the jobs that young people were able to secure were most often low paid, low skilled and temporary. In comparison to more lucrative and familiar (if also violent and dangerous) street-based forms of income generation such as drug dealing and the theft and exchange of stolen goods, these legal jobs could be experienced as much less interesting and more monotonous. In some cases, the effects of eventually losing legal jobs could be disastrous for youth, and result in re-entrenchment in the inner city drug scene. Moreover, a number of youth fell victim to “too-good-to-be-true” employment opportunities and scams that embroiled them in serious legal trouble, or, more benignly, resulted in wasted time. With regards to the latter, one young woman spent several days distributing flyers on the understanding that she would be paid by her ‘employer’ once the task was completed. However, upon completing the task she was unable to contact the employer and receive payment. With regards to the former, Ryan, a 25-year-old Caucasian man, recounted how he was connected with an ‘employer’ through a legitimate job agency, who nevertheless turned out to be a con artist. After working as a mover for two weeks, the young man attempted to cash his pay cheque, which was identified by the bank as a fraudulent cheque. After a criminal investigation into his involvement in the scam, the young man himself ended up being charged with fraud (by that point the employer had disappeared). Despite his best efforts to prove that he had no knowledge that the business he was working for was actually operating illegally, he was sentenced to jail time. As Ryan described:

*I started getting my life together. I was working a legit job. I was living a normal routine. I was startin’ to put on weight again. Everything was starting to go good. I was saving money. [pause] And then, I guess I was meant to be a homeless drug addict. Cause for some reason it just… [pause] I go to work. And the guy that I’m working for is gone. Disappeared. The job didn’t exist anymore… Because I was a drug addict and had a previous criminal record they didn’t believe me. They didn’t care. The judge said,* “We don’t believe you. People will do anything to get money for drugs.” *I even presented the fact that I was clean. Had a sponsor and everything. It didn’t matter. Like once a drug addict, in their eyes you’re always a drug addict. […] I was gonna continue working with this legit job. If this didn’t happen and I didn’t go to jail, I probably would still be clean and I probably still be working.*

After serving his prison term, Ryan returned immediately to the Downtown Eastside. With no money and no place to stay, he once again found himself homeless, and quickly began using and selling drugs. At the time of writing, he continues to live in the Downtown Eastside, albeit in supportive housing.

#### Challenges finding housing outside of Vancouver’s drug scenes

Young people also expressed a strong desire to find housing that was outside of the city’s drug scenes. Over the course of the study period, some youth managed to do so. While some of the housing that youth were able to find outside of the scene was temporary and precarious (e.g., shelters, ‘couch surfing’ in the suburbs), participants consistently described how being physically removed from Vancouver’s inner city and the associated drug scenes was critical to reducing their drug scene involvement, including reducing their drug use. These youth often described how being physically present in the inner city could ‘trigger’ their desire to engage in binge drug use, as it was more difficult to resist the urge to use drugs when substances were highly visible and easily accessible. For example, Jim, a 24-year-old Caucasian man described how securing a bed at a shelter outside of the inner city had helped him transition away from various facets of the scene, including using and dealing drugs:

*When I’m on the North Shore [a suburb of Vancouver] staying at the shelter, like, I just – I don’t know, I just don’t see anything…If I was down here, like, I’d be getting’ the cravings, like, all day, like – you know? Just seeing somebody smoking their [crack or crystal methamphetamine] pipe, you know? Like, over there [on the North Shore] I don’t have to worry about it. I don’t see anybody smoking their pipe or talking about it, you know?*

Participants’ attempts to find longer term and more permanent housing outside of Vancouver’s inner city were largely obstructed by meagre monthly social assistance (welfare) payments and the extremely high cost of housing in Greater Vancouver. Even among those who received relatively higher rates of disability social assistance (those qualifying for different levels of disability assistance can receive additional financial support,^1^ being a young person on welfare almost always automatically “marked” participants as undesirable tenants to prospective landlords. As Megan, a 22-year-old Indigenous woman described:

*I don’t know who to ask [about getting better housing], or anything. Like who to go to, to help me get a place because – I don’t know. It’s hard to get a place. Especially if you’re on welfare. You don’t get that much money on welfare. So it’s hard to find a place who, who will rent you […] to younger people, because they don’t trust them. They think they’re gonna ruin the place and that they’re gonna party too much. Or that they’re gonna use drugs there. […] People don’t like to, rent to younger people who they can’t trust.*

Young people often found themselves in a situation where the only relatively stable housing they could access was low income, government subsidized housing in Vancouver’s inner city designated for “hard to house” residents who use drugs.

For those participants who did find jobs and housing outside of Vancouver’s inner city, some described how this geographical shift in their day-to-day lives entailed leaving their existing friends and social networks behind. For many, this process was viewed as essential to successfully exiting the drug scene. For example, David, a 26-year-old Caucasian man who moved out of downtown and into a suburb of Greater Vancouver described how this was a purposeful strategy to avoid the daily activities that accompany drug use and drug scene involvement:

*I just moved to Surrey again, three days ago. A nice four bedroom…in a quieter neighbourhood, I guess, than I’m used to, but that’s okay. I’m getting older, less inclined to want to be around drama and the people that cause drama [in downtown Vancouver]…I don’t really come downtown much anymore. The environment here is kind of grimy. [There are people here] I don’t want to associate with anymore. People that aren’t going anywhere. […] This whole little part of Vancouver here. With all of the homeless and the addicts that I’ve known for years and years and years and they’re still here and they’re not doing nothing. I’m trying to do something with my life and trying to get somewhere. I can’t associate with them anymore.*

#### Challenges with addiction treatment

Reducing problematic and binge drug use was viewed as critical by youth in order to meaningfully exit the drug scene. Participants did describe services in the inner city where they could potentially get connected to drug treatment programs and detox beds (e.g., *Insite* – Vancouver’s supervised injection site, community health clinics). However, as with accessing service support in finding housing and employment, youth also expressed a sense of “not knowing where to go to find help” and “needing to take care of things for themselves.” For example, Megan described:

*I don’t have any place where I can go to get help. I know I can go to Pender Clinic or Insite to ask where, like – if I really need something, where I can go to get that. But um, besides that, I don’t have any place where I – they can help me or anything.*

A number of the participants in this study simply did not attempt to access treatment and detox services, even when these were accessible. Instead, they often attempted to reduce their drug use using their own strategies. These strategies included attempts to quit “cold turkey” and “self detox,” as well as the use of other substances (e.g., marijuana, dextromethorphan from cough syrup, crystal methamphetamine) to mediate the withdrawal symptoms of “coming off of” crack cocaine and opiates [13]. Those youth who did access or attempt to access drug treatment experienced numerous barriers, including difficulties entering methadone maintenance treatment (MMT) programs due to the cost of daily dispensing fees, long waiting lists for residential treatment programs, and aging out of State-provided youth services (which occurs at ages 21, 24 and in some cases 26 in our setting, depending on the service). Emily, a 28-year-old Caucasian woman, described the difficulties she experienced in getting on MMT and welfare – steps that she viewed as crucial to fully exiting the scene and moving to Quebec (a province in Eastern Canada) to live with her mother. Her story revealed a complex set of actions that were required in order for her to access MMT and welfare in the context of homelessness and addiction. Namely, in order to have the dispensing fees for methadone covered by the government (fees she could not afford in the context of a severe addiction to opiates and homelessness), she needed to qualify for and receive social assistance (welfare). In order to qualify for social assistance, she needed to provide a legal address, and demonstrate that she could not work as a result of her addiction to heroin. Like many young people in our setting, Emily was living and sleeping outside at this time, rather than in a room in a run down and potentially dangerous single room occupancy hotel:

*I tried so many times to get on welfare, but I didn’t have an address, and I told them all, “Well, I’m homeless,” and they’d say,* “Well, you’re healthy, so go get a job.” *And I’d be like,* “Well, you know, I’m on drugs.” *“Well, then go get clean.”* “Well, that’s why I’m here. I’m trying to get on methadone.” *You know, and that was like the Catch-22, like that – I’d have to be on methadone [to qualify for welfare], and then that’s how I finally got on welfare, ‘cause I got on methadone.*

Emily was ultimately able to get on social assistance and methadone, largely as a result of the support of her father who initially paid the dispensing fees for MMT and allowed her to use his address for the purposes of securing welfare. He also eventually allowed her to stay at his house while she was transitioning off of heroin and onto methadone. At the time of an interview in 2012, Emily had not used heroin for one year:

*I’m lucky because most of the people who live out here [on the streets], their parents don’t even live out here [in Vancouver]. They have no connections with their parents … if it wasn’t for my dad, I still wouldn’t even be on methadone because he paid 15 dollars a day [dispensing fees] for me to do so. Yeah, I mean he gave me a place to stay, so. I may not be clean without him –* ‘cause then a lot of people too, I know, would go to detox and get clean, and then come out and they don’*t have a house, so they’re right back on the street with us, and it’s like, what are you going to do? Just sit there and not – everyone’s just like back in the same routine, right? Yeah, I was lucky I had my dad to go to.*

For Emily and a few other young people, having access to social networks (e.g., family, friends) outside of the drug scene was a critical factor in reducing their drug use, as well as finding housing and obtaining financial support. It should be noted, however, that for the vast majority of participants these kinds of supports were not an option – they did not feel that they could appeal to family members for help (if they knew how to contact them at all), nor did they feel that they could appeal to old friends and acquaintances from outside of the scene.

## Discussion

Despite expressing a desire and strong motivation to “get out”, almost all of the participants were unable to leave the inner-city drug scene for sustained periods of time. Among those who successfully transitioned their housing or income-generation activities outside of the inner-city drug scene, as well as those who reduced their drug use, their transitions were usually partial and temporary. For example, while a small sub-set of our participants managed to move home, stay with family or friends, reduce their drug use and/or find what they considered to be “legitimate” forms of employment, it became clear that many *other* facets of their daily lives remained firmly established and interconnected within Vancouver’s inner-city drug scene. As a result, it was extremely difficult for young people to enact long-term change without multiple forms of support across the various facets of their lives that were entrenched in the scene, including their income-generation activities, social networks and housing. For example, as youth sought out addiction treatment services (e.g., methadone) to support cessation or reduction of harmful forms of drug consumption, participants reported both administrative and financial barriers to engaging with these services in ways that could also concurrently or successively facilitate the reduction of their involvement with *other* facets of the scene, including safe housing and employment or training opportunities that exist outside of the scene.

Some, but very few, managed to “get out and stay out” of the inner-city drug scene. Finding housing and/or employment, and reducing problematic drug use via drug treatment or other means were all critical moments in youths’ exiting trajectories. For example, a small subset of youth managed to move away from the inner city, usually into one of the suburbs of Greater Vancouver where housing is more affordable. Nonetheless, as we learned about how their experiences with exiting the scene actually unfolded over time it became increasingly clear that they faced a synergistic set of barriers in their efforts to both “get out” of the scene and “get into” a new life free from involvement with the social and physical features associated with the urban drug scene. For instance, as youth attempted to “get into” new lives out of the scene (e.g., physically and socially), accessing housing, employment or other forms of assistance (e.g., income assistance, drug treatment) were often complicated by a dearth of social (e.g., friends, family) or cultural (e.g., education, skills, appearance) capital that could provide them with support in navigating highly complex bureaucratic health and social service systems that were situated in so-called “mainstream society”. For example, due to the limited exposure street-entrenched youth often have with so-called “mainstream society”, many are unable to identify what might constitute a “too-good-to-be-true” employment or income-generation opportunity (e.g., scams).

These findings highlight the limitations of conventional social and health services available to support youth with reducing their involvement with Vancouver’s inner-city drug scene, despite expressing a strong desire and engaging in their best efforts to do so. In fact, very few youth reported engaging with support services in ways that either helped them find housing or get a job outside of the scene, or to reduce harmful forms of drug consumption – and, none of the youth reported receiving assistance that could provide *holistic* support (i.e., support that addresses the interconnected facets of a young person’s involvement in the scene) in addressing their aims either concurrently or consecutively. Indeed, while some services are available to support youth with income assistance or finding a job in Vancouver’s inner city, most of our participants were either entirely unaware of the existing support services available to them in Vancouver’s inner city or expressed a strong reluctance to engaging with support due to a mistrust of “the system”. Almost all of our participants also insisted the steps they needed to take to exit the scene needed to be done on their own. As such, our findings align with previous research examining young people’s experiences with ‘moving out’ of homelessness [22] in which young people attempting to exit the inner-city drug scene expressed favour towards ‘independent’ exits from homelessness (e.g., transition into private housing without the support of formal social services) over those that were characterized as ‘dependent’ exits (e.g., transitional steps towards exiting, including staying with family or friends) from homelessness.

Optimizing the best strategies for youth entrenched in inner-city drug scenes at the appropriate time in the life course is integral to improving young people’s opportunities to exit the scene. Our findings underscore the need to enhance the provision of holistic programs and services that support youth entrenched in inner-city drug scenes while concurrently transitioning multiple and interconnected facets of their lives *into* a new routine and life that is no longer firmly established within the inner-city scene. This approach to intervention requires a dynamic process of information exchange, policy development, and program-planning activities that adapt and respond to key transitional periods (e.g., contemplation), as well as critical moments in which young people are actively seeking out ways to transition out of the scene.

There are examples of promising interventions in this area that may leverage opportunities to provide more holistic and/or low-threshold services. For example, providing support opportunities to *combine* and *enhance* existing approaches to acquiring income assistance, educational and/or occupational and other life skills training opportunities and support (e.g., Street Health’s ID Safe – safe storage services of important ID for homeless or under-housed people), ‘low threshold’ addiction treatment services (e.g., Vancouver’s Portland Hotel Society’s OnSite Detox & Transitional Housing Program), and supportive housing outside of the scene (e.g., Vancouver’s MPA Society’s programs and services that aim to offer residents full participation in society in neighbourhoods throughout Metro Vancouver) represents promising opportunities to influence youths’ exiting trajectories. Moreover, in the Province of BC, new evidence-based regulatory policies and clinical reforms are opening up novel opportunities to address problematic substance use, including innovative pharmacotherapeutic approaches, new and revised clinical practice guidelines, dedicated detox and treatment beds for young people, as well as the integration of addictions medicine and care into other areas of the health service delivery system (e.g., community-based care). For example, in response to the current fentanyl and opioid overdose crisis in Vancouver, along with the growing body of evidence supporting the effectiveness of Suboxone®, the Vancouver Coastal Health Authority (VCH) issued new clinical addiction treatment guidelines in 2015 that make Suboxone® a recommended first-line pharmacotherapy for opioid use disorder among young people. While our data do not have the capacity to distil the extent to which these new and emerging ‘low-threshold’ pharmacotherapies will influence young people’s exiting trajectories over time, these policy changes may represent a critical ‘next step’ for improving young people’s opportunities to access care and exit the scene.

Significant challenges remain, however, as these findings also reveal youth’s affinity for exiting independently – i.e., accomplishing many of their transitions out of the scene on their own and without the support of formal services. Indeed, these findings underscore the need for intervention planners to adapt approaches that can account for these complexities, including multi-faceted “upstream” policy responses that reunite “harm reduction” with direct adaptation of the larger systems of power and political economy that create harm, and taking seriously the need for larger scale structural change that prevent youth from “entering” urban drug scenes in the first place [23–26]. Indeed, socially just approaches to reduce human suffering among young people entrenched in Vancouver’s urban drug scene will require inter-sectoral approaches and sustained commitment from a variety of stakeholders (e.g., political, public health, law enforcement).

### Strengths and limitations

This study has several strengths and limitations. First, while the composition of our sample is meant to reflect the diversity of experiences and backgrounds of youth involved within Vancouver’s urban drug scene, the youth in our sample includes many of the ‘higher’ risk youth involved with Vancouver’s urban drug scene – particularly, a group of young people who are deeply entrenched within Vancouver’s inner-city drug scene [13]. As such, we acknowledge that our sample is not representative of all youth in the inner-city drug scene. Second, while many of these findings may have implications for youth living in urban drug scenes elsewhere, due to the unique social and spatial features of Vancouver’s urban drug scene (e.g., features of the social service infrastructure, socio-legal context), there are some findings that may not be generalizable elsewhere. Nevertheless, by drawing on longitudinal ethnographic techniques, our study was able to identify how young people plan to “exit” an urban drug scene, as well as how their “exiting” trajectories actually unfold over time.

## Conclusion

While young people entrenched in Vancouver’s inner-city drug scene express a strong desire to – often *independently* – exit the scene, they face a set of key challenges and barriers, including securing legitimate employment or training, distancing themselves (both physically and socially) from the urban drug scene, and reducing their drug consumption. Taken as a whole, these findings highlight a set of social and structural barriers that impede young people’s capacity to meaningfully distance themselves from Vancouver’s urban drug scene, as well as the limitations of conventional social and health services available to support youth to reduce their engagement with inner-city risk environments. Integrated policies and programing interventions that can facilitate young people’s efforts to prevent and/or reduce engagement with urban drug scenes are critically needed, including a holistic and adaptive approach towards educational and/or occupational training opportunities, ‘low threshold’ addiction treatment services, as well as access to supportive housing outside of the scene.

## Abbreviations

ARYS: At-Risk Youth Study
ID: Identification
Meth: crystal methamphetamine
MMT: methadone maintenance treatment
SRO: single room occupancy hotel

## Footnotes

1 In British Columbia, youth who receive Disability 2 assistance or meet the Persons with Persistent Multiple Barriers (PPMB) criteria are entitled to an additional amount of approximately five and three hundred dollars respectively. Both support levels also provide individuals with an additional fixed monthly rent of CAD$375 for rooms in Single Room Occupancy Hotels or supportive housing buildings which is deducted directly from social assistance payments.
